# Antifungal Susceptibility and Resistance-Associated Gene Expression in Nosocomial *Candida* Isolates

**DOI:** 10.3390/jof11120895

**Published:** 2025-12-18

**Authors:** Fabiola Berenice Hernandez-Reyes, Luis Alfonso Muñoz-Miranda, Manuel R. Kirchmayr, Pablo César Ortiz-Lazareno, Rafael Cortés-Zárate, Maricarmen Iñiguez-Moreno, Heriberto Jacobo-Cuevas, Cesar Arturo Nava-Valdivia

**Affiliations:** 1 Programa de Maestría en Microbiología Médica, Departamento de Microbiología y Patología, Centro Universitario de Ciencias de la Salud, Universidad de Guadalajara, Guadalajara 44340, Jalisco, Mexico; fabiola.hernandez8654@alumnos.udg.mx; 2 Centro de Investigación en Enfermedades Infectocontagiosas, Departamento de Microbiología y Patología, Centro Universitario de Ciencias de la Salud, Universidad de Guadalajara, Guadalajara 44340, Jalisco, Mexico; 3 Unidad de Biotecnología Industrial, Centro de Investigación y Asistencia en Tecnología y Diseño del Estado de Jalisco A.C. (CIATEJ), Subsede Zapopan, Zapopan 45019, Jalisco, Mexico; 4 Centro de Investigación Biomédica de Occidente, División de Inmunología, Instituto Mexicano del Seguro Social (IMSS), Guadalajara 44340, Jalisco, Mexico; 5 Department of Physics and Mathematics, School of Engineering and Technology, Universidad de Monterrey, San Pedro Garza García 66238, Nuevo León, Mexico; 6 Programa de Posdoctorado, Departamento de Psicología Básica, Centro Universitario de Ciencias de la Salud, Universidad de Guadalajara, Guadalajara 44340, Jalisco, Mexico

**Keywords:** *Candida* species, antifungal susceptibility, gene expression

## Abstract

**Background:** Nosocomial infections represent a significant clinical burden due to high morbidity, mortality and healthcare costs. Invasive fungal infections, particularly those caused by *Candida* species, are of growing concern due to increasing antifungal resistance, which limits therapeutic options and worsens patient outcomes. This study aimed to characterize the prevalence, species distribution, antifungal susceptibility profiles, and molecular mechanisms of resistance in clinical *Candida* isolates from hospitalized patients. **Methods:** A cross-sectional study was conducted involving 55 hospitalized patients, yielding 60 isolates from blood, secretions, fluids, and catheter tips. Species identification was performed using chromogenic media and confirmed by MALDI-TOF MS. Antifungal susceptibility testing followed CLSI M27-A4 broth microdilution guidelines for amphotericin B, fluconazole and 5-flucytosine. Gene expression of *ERG2*, *ERG11* and *MDR1* was evaluated by RT-qPCR after exposure to subinhibitory antifungal concentrations using the 2^−∆∆Ct^ method. **Results:** *Candida albicans* was the most frequent species, followed by *Nakaseomyces glabratus*, *C. tropicalis* and *C. parapsilosis*. Resistance varied among species, with elevated rates for fluconazole. *ERG2* was notably overexpressed in amphotericin B-resistant isolates, while *ERG11* and *MDR1* showed species-dependent variation. **Conclusions:** Resistance mechanisms in *Candida* are species-specific and drug-dependent. Accurate species identification and understanding their molecular profiles are essential to guide targeted antifungal therapy and improve clinical outcomes.

## 1. Introduction

Nosocomial infections are defined as those that are neither present nor detectable at the time of hospital admission but develop after at least 48 h of hospitalization [1]. Among these, invasive fungal infections represent a major clinical challenge, significantly increasing morbidity and mortality in hospitalized, immunocompromised, pediatric, and elderly patients, while also imposing a substantial economic burden on healthcare systems worldwide [2]. Mortality rates as high as 60% have been reported, with yeasts of the genus *Candida* ranking as the fourth most common agents responsible for nosocomial septicemia [3,4].

*Candida* spp. are commensal organisms colonizing mucosal surfaces, including the oral, vaginal, gastrointestinal, and skin areas. However, under certain predisposing conditions such as antibiotic therapy, indwelling catheters, surgical interventions, neutropenia, parenteral nutrition, and comorbidities including hematological malignancies, cancer, renal, pulmonary, neurological, gastrointestinal, and hepatic disorders, as well as HIV and diabetes mellitus, they can shift from harmless colonizers to cause superficial or systemic infections [5].

Accurate species-level identification of *Candida* is critical for patient management and guiding therapy. Novel molecular approaches, such as PCR-based diagnostics and mass spectrometry, now allow rapid detection and characterization of *Candida* species, improving clinical decision-making and enabling real-time epidemiological surveillance [6,7].

The limited number of unique fungal targets and the emergence of resistance challenge the treatment of candidemias. Currently, four main classes of antifungal agents, echinocandins, polyenes, azoles and pyrimidine analogs, are approved for clinical use, yet their effectiveness is increasingly compromised by multidrug-resistant *Candida* isolates [8,9].

Amphotericin B (AMB) is a polyene that binds to ergosterol in the fungal membrane, forming pores that compromise membrane integrity and induce cell death; reduced susceptibility has been associated with mutations in *ERG2*, *ERG3*, and *ERG6* (encoding enzymes in ergosterol biosynthesis), which alter membrane composition and can affect virulence and treatment outcomes [10]. Also, fluconazole (FLZ), a widely used azole, inhibits the product of the *ERG11* gene (encoding lanosterol 14α-demethylase), disrupting ergosterol biosynthesis and leading to accumulation of toxic sterol intermediates. Resistance can develop through *ERG11* mutations or overexpression and via efflux pumps such as the *MDR1* gene (encoding multidrug transporter), reducing drug susceptibility and limiting clinical efficacy [11]. Finally, flucytosine (5-FC) differs mechanistically as a nucleic acid analog, entering the cell through cytosine permease (*FCY2)* and being converted to 5-fluorouracil by the product of the *FCY1* gene (encoding cytosine deaminase), thereby interfering with RNA and DNA synthesis; mutations in *FCY1* confer rapid resistance under monotherapy, highlighting the importance of combination therapy [12].

Gene expression analysis and other molecular profiling approaches have become a powerful tool for elucidating fungal adaptive responses to antifungal agents. Assessing the expression of antifungal resistance–related genes is crucial for uncovering the molecular mechanisms underlying reduced susceptibility. These approaches provide a detailed understanding of the genetic determinants underlying resistance, thereby offering opportunities for early detection, improved diagnostics, and personalized antifungal therapy [13].

These mechanisms collectively highlight how specific genetic alterations mediate antifungal resistance, shaping clinical efficacy and biological fitness in pathogenic *Candida* species. The increasing resistance represents a global health threat. The WHO 2022 Priority Fungal Pathogens list highlights *Candida auris* and *Candida albicans* as critical priority pathogens, while *Pichia kudriavzevii* (formerly *Candida krusei*), *Nakaseomyces glabratus* (formerly *Candida glabrata*), *Candida parapsilosis*, and *Candida tropicalis* are designated as medium to high priority [14].

The present study aims to investigate the molecular basis of antifungal resistance in clinical *Candida* isolates by integrating species-level identification, susceptibility profiling, and expression analysis of key resistance genes (*ERG2*, *ERG11*, *MDR1*) to elucidate mechanisms underlying therapeutic failure in nosocomial infections.

## 2. Materials and Methods

### 2.1. Study Design

Cross-sectional study.

### 2.2. Clinical Setting and Laboratory Assessment

The research was conducted in accordance with the Declaration of Helsinki (Helsinki, Finland, 2024) and approved by the Local Health Research Committee (Comité Local de Investigación en Salud 1305; approval code R-2024-1305-045).

This study included 55 hospitalized patients confirmed by yeast infections, as evidenced by positive culture results. An infection was defined as the initial isolation of *Candida* spp. or related species from a clinical specimen obtained ≥48 h after hospital admission. Mixed infection was defined as the presence of more than one *Candida* species within the same patient. All clinical specimens and data from hospitalized patients were handled according to biosafety standards and Mexican data protection laws.

Sociodemographic and clinical data, including sex, age, body mass index, comorbidities, clinical diagnosis, specimen origin, pharmacological treatments, and requesting hospital service (Internal Medicine, Nephrology, General Surgery, or Gynecology), were retrieved from electronic medical records.

### 2.3. Samples Collection Isolates

Sixty *Candida* strains from different nosocomial clinical isolates corresponding to 55 patients were analyzed between September 2024 and February 2025. Clinical yeast isolates were obtained from a variety of biological specimens, including peritoneal, ascitic, and cerebrospinal fluids, blood, intravascular catheters, abscesses, wound exudates and other secretions, but were not included respiratory isolates. The isolates were obtained through conventional microbiological methods using Sabouraud dextrose agar as the primary culture medium. All specimens were collected from hospitalized patients at the General Hospital No. 14, Mexican Social Security Institute (Instituto Mexicano del Seguro Social, IMSS), Guadalajara, Jalisco, Mexico.

### 2.4. Strains Identification of Candida Species

Isolates were thawed and plated on CHROMagar *Candida* (Becton Dickinson, BD^®^, Franklin Lakes, NJ, USA) and incubated at 37 °C for 48 h. Presumptive species identification was based on colony morphology and chromogenic characteristics.

For definitive identification, colonies were subcultured onto Sabouraud Dextrose Agar (Becton Dickinson, BD^®^, USA) and incubated at 37 °C for 24–48 h. A single colony was transferred to a steel target plate, treated with 1 µL of formic acid, and air-dried before adding of 1 µL of α-cyano-4-hydroxycinnamic acid (HCCA) matrix solution. After drying, matrix-assisted laser desorption/ionization time-of-flight mass spectrometry (MALDI-TOF MS) was performed using the MicroFlex LT^®^ system (Bruker Daltonics, Bremen, Germany) with the MBT_FC method, acquiring six replicate spectra per spot. The resulting protein profiles were compared against the BDAL v.10^®^ reference database (Bruker Daltonics, Bremen, Germany), and species-level identification was considered reliable at a log score ≥ 1.7, enabling accurate taxonomic classification based on proteomic fingerprints [15].

### 2.5. Antifungal Susceptibility Testing (AFST)

Antifungal susceptibility was performed using the broth microdilution method according to the Clinical and Laboratory Standards Institute (CLSI) reference protocol for yeasts, M27-A4 [16], with RPMI 1640 medium (Sigma-Aldrich^®^, St. Louis, MO, USA) buffered with MOPS and supplemented with 0.2% glucose. Following species confirmation by MALDI-TOF MS, isolates were subcultured on Sabouraud Dextrose Agar (Becton Dickinson, BD^®^, USA) and incubated at 37 °C for 48 h. Yeast suspensions were prepared to a final density of 2.5 × 10^3^ CFU/mL (target range: 1–5 × 10^3^ CFU/mL), in accordance with CLSI recommendations. Plates were incubated at 35 °C for 24–48 h.

The antifungal agents tested included amphotericin B (Sigma-Aldrich^®^, St. Louis, MO, USA), fluconazole (Sigma-Aldrich^®^, St. Louis, MO, USA) and 5-flucytosine (Sigma-Aldrich^®^, St. Louis, MO, USA). AMB, a water-insoluble agent, was prepared in DMSO with a final range of 0.03–16 µg/mL, while, for water-soluble compounds (FLZ and 5-FC), final concentration ranges of 0.12–64 µg/mL were prepared in RPMI 1640/MOPS/0.2% glucose. Sterility controls and growth controls contained the standardized yeast inoculum without antifungal agents. Both controls were included in spectrophotometric readings to validate assay performance. To determine the antifungal susceptibility profile, all samples were analyzed in both biological duplicates

Isolates were classified as susceptible (S), intermediate (I), susceptible dose-dependent (SDD), or resistant (R) according to the Clinical and Laboratory Standards Institute (CLSI) M27-A4 and M60 using spectrophotometric measurement at a wavelength of 405 nm with agitation. MIC_50_, MIC_90_, geometric mean MIC, and MIC ranges were calculated to summarize antifungal susceptibility profiles [16,17,18].

### 2.6. Inducible Expression of Resistance-Related Genes Under Antifungal Exposure

*Candida* isolates exhibiting MIC ≥ 64 µg/mL for fluconazole or MIC ≥ 16 µg/mL for amphotericin B were selected. One representative strain was randomly chosen for inducible expression of resistance-related genes (*ERG2*, *ERG11* and *MDR1*) under antifungal exposure.

To assess inducible gene expression, cultures were initially adjusted to 1 × 10^6^ cells/mL in 5 mL of SDB and incubated at 30 °C with agitation (160 rpm). After 4 h, an aliquot of 250 µL was collected as untreated control (drug-free). Following this, the cultures were exposed to a subinhibitory concentrations of FLZ or AMB (one dilution below the MIC of each isolate). Incubation continued for 7 h on antifungal exposure according to the method described by Paul et al., 2022 [19].

### 2.7. RNA Extraction

Total RNA was extracted from *Candida* spp. cultures grown in Sabouraud Dextrose Broth (SDB) during logarithmic growth using a modified TRIzol™ RNA (Invitrogen, Carlsbad, CA, USA) extraction method as described by Chomczynski (1993) adapted with glass beads [20,21,22]. Extracted RNA was further treated with RNase-free DNase I (Qiagen, Hilden, Germany) to remove genomic DNA contamination. RNA quality and concentration were assessed using a NanoDrop spectrophotometer (Thermo Scientific, Waltham, MA, USA), with 260/280 ratios ranging from 1.85 to 2.1. RNA integrity was confirmed by 1% agarose gel electrophoresis containing 6% sodium hypochlorite.

### 2.8. Reverse Transcription Protocol

High-quality RNA samples were subsequently used for cDNA synthesis with the Verso cDNA Synthesis Kit (Thermo Fisher Scientific, MA, USA), using 1 µg of RNA template on a MiniAmp Plus Thermal Cycler (Thermo Fisher Scientific, MA, USA). Reverse transcription was performed at 42 °C for 30 min, followed by inactivation at 95 °C for 2 min. All procedures were performed according to the manufacturer’s instructions.

### 2.9. Primer Design for Antifungal Resistance Genes

Drug-induced expression of target genes, including *ERG2* and *ERG11* (involved in ergosterol synthesis) and *MDR1* (drug efflux transporter), was evaluated. Primers were either obtained from previously published studies or custom-designed using BLAST(http://www.ncbi.nlm.nih.gov/tools/primer-blast, accessed on 23 July 2025) Primer Design and IDT OligoAnalyzer (https://www.idtdna.com/pages/tools/oligoanalyzer, accessed on 23 July 2025). Full primer sequences are listed in Supplementary Table S1.

### 2.10. Expression Analysis of Target Genes

Relative expression of the genes was assessed by RT-qPCR using 25 ng/µL of cDNA per reaction and 0.3 using µM of each primer pair. Each of the sample assays with and without antifungal exposure was performed with identical duplicate replicates on a StepOne Real-Time PCR system (Applied Biosystems, Foster City, CA, USA) using Maxima SYBR Green Master Mix (Thermo Fisher Scientific, MA, USA), following the manufacturer’s instructions. All samples were analyzed in biological and technical duplicate, with a no-template control incorporated for each primer pair. The amplification program consisted of an initial denaturation at 95 °C for 10 min, followed by 40 cycles of denaturation at 95 °C for 15 s, annealing and extension at 60 °C for 1 min. A melting curve analysis was performed at the end of the program to verify product specificity. Gene expression levels were calculated relative to untreated controls using the 2^−ΔΔCT^ method, with *β-actin* (*ACT1*) as a stable reference gene for normalization [23].

### 2.11. Statistical Analysis

Qualitative variables are expressed as frequencies and percentages, while quantitative variables are summarized using measures of central tendency and dispersion measures. Comparisons between groups were conducted using Student’s t-test for quantitative variables and Chi-square or Fisher’s exact test for qualitative variables. Antifungal susceptibility, including minimum inhibitory concentrations (MICs), was interpreted according to CLSI guidelines. Relative gene expression (fold change) was calculated using the 2^−ΔΔCt^ method with *β-actin* as the internal reference gene. Statistical significance was *p* ≤ 0.05, with a 95% confidence level and 80% statistical power. Analyses were performed using SPSS v.26 (IBM Corp., Armonk, NY, USA) and GraphPad Prism v.8.00 (San Diego, CA, USA).

## 3. Results

### 3.1. Sociodemographic and Clinical Characteristics

The study included 55 hospitalized patients, of whom 61.8% were male and 38.2% female, with a mean age of 59 years and a mean body mass index (BMI) of 27.6 kg/m^2^. Diabetes mellitus was the most common comorbidity (40%), and chronic kidney disease was the most frequent specific diagnosis (32.7%). The highest proportion of *Candida* isolates came from the Internal Medicine service (45.5%), and secretions were the most common specimen type (49.1%). None of the patients had received antifungal treatment at the time of sample collection [Table 1].

### 3.2. Phenotypic and Taxonomic Identification of Candida

**Figure 1:**
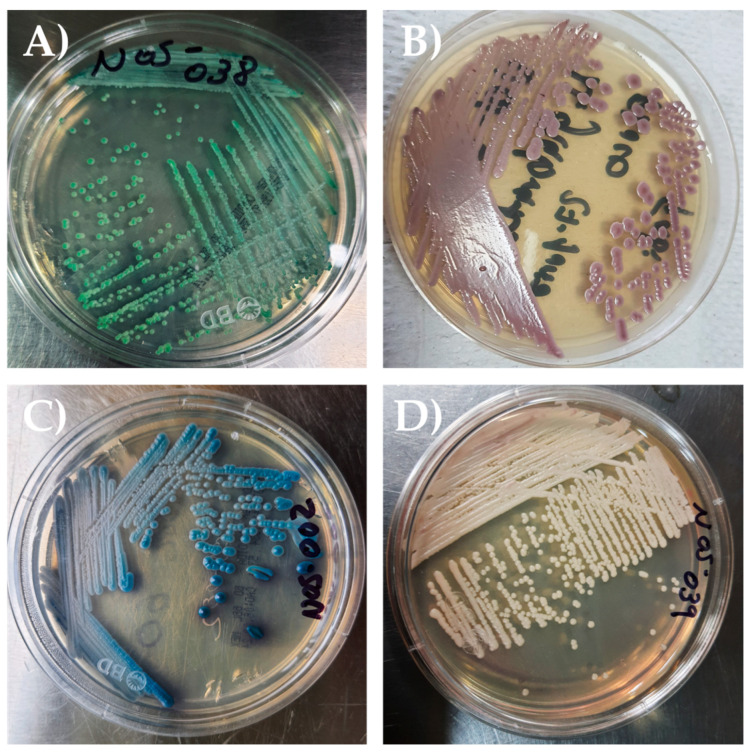
Yeast species were presumptively differentiated on CHROMagar *Candida*™ based on colony chromogenic characteristics. (**A**) *Candida albicans*. (**B**) *Nakaseomyces glabratus*. (**C**) *Candida tropicalis*. (**D**) *Candida* spp.

Initially regarding presumptive phenotypic identification, CHROMagar Candida™ (Paris, France) was employed to screen *Candida* isolates and to determine whether more than one species was present in a single sample. Sixty isolates were recovered from 55 patients [Figure 1].

In our study, based on chromogenic agar identification, the most prevalent species was *Candida albicans* (36.7%), followed by *Candida tropicalis* (18.3%), *Nakaseomyces glabratus* (formerly *Candida glabrata*) (16.7%), and *Pichia kudriavzevii* (3.3%). Isolates that could not be identified at the species level were classified as *Candida* spp. (25%).

**Figure 2:**
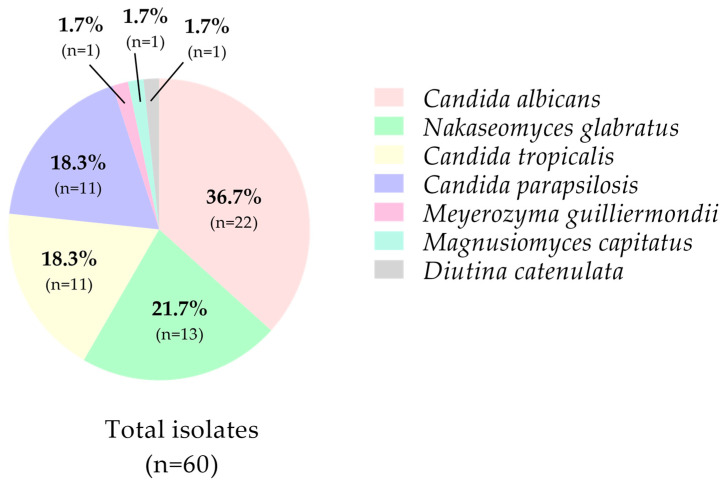
Species prevalence of clinical *Candida* isolates identified by MALDI-TOF MS.

Subsequently, taxonomic identification of the 60 isolates was confirmed by MALDI-TOF MS™. Among the isolates, *Candida albicans* was the most prevalent species (36.7%), followed by *Nakaseomyces glabratus* (21.7%), *Candida tropicalis* and *Candida parapsilosis*, each representing 18.3%. Less common species included *Meyerozyma guilliermondii*, *Magnusiomyces capitatus*, and *Diutina catenulata*, each accounting for 1.7% of the isolates [Figure 2].

Males contributed the majority of isolates (61.8%). *C. albicans* was the leading species, followed by *C. parapsilosis* (20.0%) and both *C. tropicalis* and *N. glabratus* (18.2% each).

Most isolates were recovered from patients in the overweight (45.5%) and normal weight (30.9%) categories. *C. albicans* was the most prevalent species across all BMI groups, with the highest proportion observed in overweight individuals (47.6%). *C. parapsilosis* was most frequently identified in normal-weight patients (54.5%), whereas *C. tropicalis* and *N. glabratus* exhibited a more balanced distribution between normal weight and overweight groups. Rare species were recovered sporadically across BMI categories.

**Figure 3:**
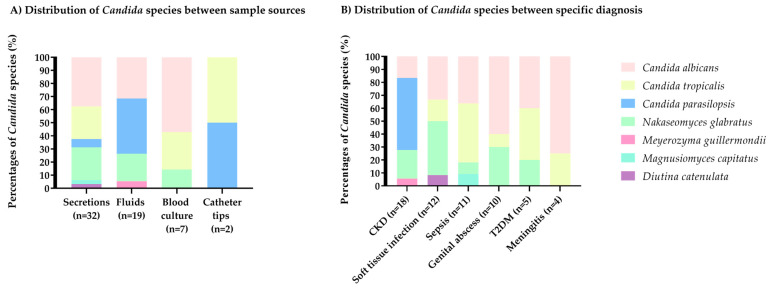
Comparison of *Candida* species between sample sources and specific diagnosis in nosocomial isolates. (**A**) Distribution of *Candida* species between sample sources. (**B**) Distribution of *Candida* species between specific diagnosis.

We observed *Candida* and related yeast species distribution revealed notable variations both across hospital departments and specimen types. Overall, *C. albicans* was the most prevalent species (38.2%), predominantly isolated in Internal Medicine and mainly from secretions (52.4%), fluids (28.6%), and blood cultures (19.0%). In contrast, *C. tropicalis* (18.2%) was most frequent in Internal Medicine and General Surgery, primarily recovered from secretions (70.0%) and blood cultures (20.0%). Similarly, *N. glabratus* (18.2%) showed a more even distribution across departments, with higher prevalence in Gynecology, and was most commonly isolated from secretions (50.0%) and fluids (40.0%), while also being recovered from blood cultures (10.0%). Conversely, *C. parapsilosis* (20.0%) was markedly predominant in Nephrology and fluids (72.7%), followed by catheters (9.1%), with no recovery from blood cultures. Furthermore, rare species, *M. guilliermondii*, *M. capitatus*, and *D. catenulata*, each accounting for 1.7% of isolates, were found exclusively in specific settings: *M. guilliermondii* in Nephrology fluids, and both *M. capitatus* and *D. catenulata* in Internal Medicine secretions [Figure 3].

Diabetes was identified in 54.5% of cases, most frequently associated with *C. albicans* (30.0%), *C. tropicalis* (23.3%), and *N. glabratus* (20.0%). Chronic kidney disease (27.3%) was mainly linked to *C. parapsilosis* (66.7%) and *N. glabratus* (20.0%).

### 3.3. Antifungal Susceptibility

Our findings revealed distinct antifungal susceptibility patterns among *Candida* species, underscoring marked variability across antifungal classes. Overall, *C. albicans* exhibited low MICs for AMB (0.5–16 µg/mL) and 5-FC (0.125–4 µg/mL), although high resistance rates were observed for FLZ in 90.9% (2–64 µg/mL). In contrast, *C. tropicalis* displayed markedly elevated FLZ MICs (0.5–64 µg/mL) with 90.9% resistance, in addition to substantial resistance to AMB (63.9%). Notably, *N. glabratus* exhibited elevated FLZ MICs (8–64 µg/mL), reflecting a partial intrinsic resistance to azoles, particularly FLZ. Meanwhile, *C. parapsilosis* demonstrated notable resistance to AMB (63.9%), while retaining low MICs for FLZ and 5-FC. These findings reveal pronounced interspecies variability in antifungal susceptibility and underscore the critical importance of precise species-level identification to guide optimal antifungal therapy in accordance with CLSI M27-A4 guidelines [Table 2].

For the uncommon yeast species *M. guilliermondii*, *M. capitatus* and *D. catenulata*, MIC values demonstrated higher resistance among antifungal agents, mainly FLZ. *M. guilliermondii* exhibited MICs of 1 µg/mL for AMB, 64 µg/mL for FLZ and 0.25 µg/mL for 5-FC. *M. capitatus* showed elevated MICs of 4 µg/mL for AMB, and 64 µg/mL of FLZ, but 0.5 µg/mL for 5-FC. *D. catenulata* presented MICs of 1 µg/mL for AMB and 64 µg/mL for FLZ, and 0.25 µg/mL for 5-FC. Currently, no standardized clinical breakpoints are available for these organisms, preventing definitive classification of their susceptibility profiles [24].

### 3.4. Relative Expression Analysis of Target Genes

Five *Candida* isolates were selected for gene expression analysis based on their antifungal resistance profiles, focusing on those with the highest MICs for each drug category. For AMB, *C. albicans* (NOS-013-*Ca*) and *C. tropicalis* (NOS-063-*Ct*), recovered from blood cultures of patients with sepsis, exhibited the highest MIC values (16 μg/mL). These isolates were selected for the analysis of *ERG2* expression. FLZ-resistant isolates (MIC > 64 μg/mL) included *C. albicans* (NOS-074-*Ca*), *C. tropicalis* (NOS-092-*Ct*), and *N. glabratus* (NOS-087-*Ng*), recovered respectively from cerebrospinal fluid in a meningitis case, a central venous catheter from a septic patient, and a blood culture from a diabetic patient with cardiac disease. These strains, which exhibited the highest azole resistance levels, were selected to evaluate *ERG11* and *MDR1* gene expression.

**Figure 4:**
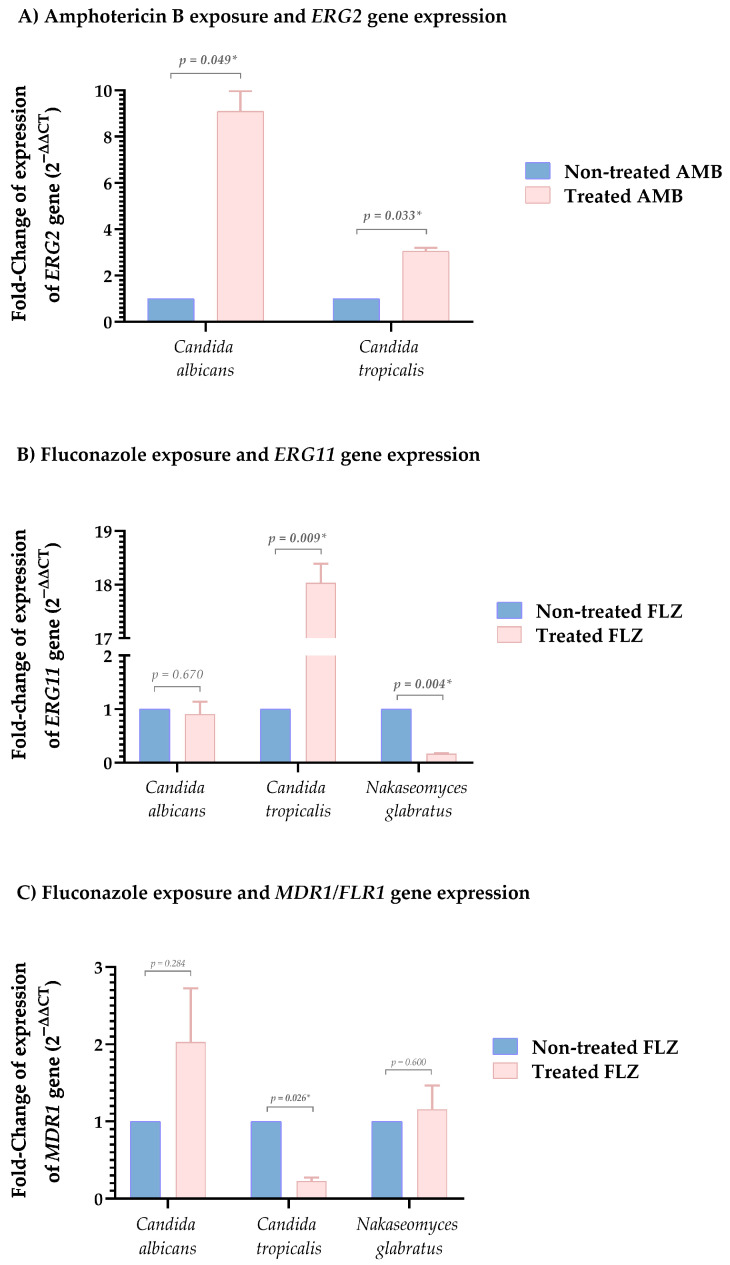
Comparison of fold-changes in antifungal resistance–associated gene expression among *Candida* species under antifungal exposure. (**A**) Amphotericin B exposure and *ERG2* gene expression. (**B**) Fluconazole exposure and *ERG11* gene expression. (**C**) Fluconazole exposure and *MDR1* gene expression. * The *FLR1* ortholog was identified in *Nakaseomyces glabratus*.

Gene expression analysis showed that AMB-resistant *C. albicans* and *C. tropicalis* significantly overexpressed *ERG2* gene (9.085 ± 0.889, *p* = 0.049; and 3.044 ± 0.149, *p* = 0.033, respectively), suggesting ergosterol biosynthesis alterations as a polyene resistance mechanism. Among FLZ-resistant isolates, *ERG11* expression varied, *C. tropicalis* showed strong upregulation (18.034 ± 0.354, *p* = 0.009), *C. albicans* remained stable (0.907 ± 0.231, *p* = 0.670) and *N. glabratus* decreased significantly (0.167 ± 0.007, *p* = 0.004), reflecting species-specific azole adaptation. Efflux pump analysis revealed moderate *MDR1* overexpression in *C. albicans* (2.030 ± 0.697, *p* = 0.284), reduced expression in *C. tropicalis* (0.229 ± 0.045, *p* = 0.026), and stable *FLR1* expression in *N. glabratus* (1.158 ± 0.308, *p* = 0.600) [Figure 4].

## 4. Discussion

*Candida* species were recovered from a wide range of biological samples in this study, reflecting their broad distribution across hospital settings. Almost half of the isolates were obtained from secretions (abscesses, ulcers, surgical wounds), over one-third from body fluids (cerebrospinal, peritoneal dialysis, ascitic), and a smaller fraction from blood. Most culture requests came from Internal Medicine, followed by Nephrology and General Surgery, underscoring the widespread impact of invasive candidiasis in different hospital services.

In contrast to our findings, hospital studies report that *Candida* spp. were isolated from various clinical samples, with bloodstream infections mainly occurring in ICUs and respiratory isolates frequently detected in mechanically ventilated patients. Species distribution varies across wards: *C. albicans* predominates in Surgical Wards and General Medicine, *C. tropicalis* and *C. parapsilosis* are common in ICUs and pediatric services, while *N. glabratus* is more frequent among elderly or oncology patients [25]. Invasive procedures and prior exposure to broad-spectrum antibiotics or corticosteroids are major infection determinants, influencing both species distribution and clinical outcomes, and highlighting the need for individualized risk assessment in hospital settings [26,27].

Clinical factors critically influence the risk of nosocomial candidiasis. In this study, *Candida* isolates were more frequent in males and in overweight or obese patients, as described in Table 1. According to reports from other populations, advanced age, high BMI, and male sex increase susceptibility, while comorbidities such as diabetes, malignancy, chronic kidney disease, and prolonged ICU stay remain major risk factors [28,29]. Type 2 diabetes increases the likelihood of invasive fungal infections 1.38-fold, particularly in patients with hyperglycemia, renal impairment, anemia, or hypoalbuminemia, aligning with our observations [30].

In this study, *C. albicans* was the most frequently isolated species, followed by *N. glabratus*, *C. tropicalis*, and *C. parapsilosis*. Globally, the epidemiology of nosocomial candidiasis is evolving, while *C. albicans* remain predominant, non-*albicans Candida* (NAC) species are increasingly implicated in bloodstream and invasive infections [31]. Large surveillance programs such as SENTRY and ARTEMIS have documented notable geographic variation, with *C. tropicalis* and *C. parapsilosis* showing particularly high prevalence in Latin America [25,32,33].

In Mexico, global trends are reflected with distinct local features. While *C. albicans* remains the leading cause of candidiasis, *C. tropicalis*, *C. parapsilosis*, and *N. glabratus* now account for a significant share of cases, especially in pediatric and ICU settings [34,35].

Rare species such as *M. guilliermondii*, *M. capitatus*, and *D. catenulata* accounted for only 1.7% of isolates, yet they are increasingly recognized as opportunistic pathogens in immunocompromised patients. Moreover, frequent misidentification and the limited use of species-level diagnostics hinder effective resistance management, delay outbreak detection, and conceal important epidemiological trends [36,37]. Given their variable susceptibility profiles, antifungal resistance among these species represents an important increasingly clinical challenge both globally and in Mexico [38].

All yeast isolates were tested against AMB, FLZ and 5-FC, with *ERG2*, *ERG11* and *MDR1* gene expression assessed in species with highest MICs. Resistance was found for all drugs except 5-FC, which showed full susceptibility. Resistance-related gene expression varied across *Candida* species, reflecting intrinsic susceptibility and adaptive antifungal responses.

Once considered exceptional, AMB resistance in *C. tropicalis* is increasingly reported worldwide. Mechanisms include *ERG2*, *ERG3* and *ERG11* mutations, ergosterol depletion, membrane alterations, oxidative-stress responses, efflux pump overexpression, biofilm formation, and protease activity [39,40].

This study is among the first to report high AMB resistance in Mexico, with notable reduced susceptibility among clinical isolates. Resistance was highest in *C. tropicalis* and *C. parapsilosis* (63.9%), followed by *N. glabratus* (46.2%) and *C. albicans* (22.7%), contrasting earlier local data showing full susceptibility and reflecting the global rise of polyene resistance [41]. In other Latin American populations, *C. albicans* and *C. tropicalis* isolates have also been reported with infrequently high MIC values (>16 µg/mL), which is consistent with our findings [42,43]. *N. glabratus* exemplifies intrinsic resistance through low ergosterol content and strong efflux systems, making even small MIC increases clinically relevant. Together, *C. tropicalis* and *N. glabratus* represent acquired and intrinsic resistance paradigms, highlighting the need for continuous species-specific surveillance and tailored therapy where AMB remains a first-line treatment [44].

We assessed *ERG2* expression, the gene encoding sterol C-8 isomerase, in *C. albicans* and *C. tropicalis* to examine AMB resistance, finding 9-fold and 3-fold increases, respectively. This protein is essential for ergosterol biosynthesis and membrane integrity, and changes in *ERG2* expression are strongly associated with AMB resistance. Also, antifungal exposure can upregulate *ERG2* and related genes as a conserved mechanism to enhance fungal survival under stress [45]. In addition, *ERG2* overexpression in dispersed *C. auris* cells following biofilm formation underscores its role in adaptive resistance, with resistant strains exhibiting over threefold higher expression than susceptible ones. This pattern, also observed in other *Candida* species, suggests a conserved mechanism that may act synergistically with additional pathways under polyene stress [46]. Loss of *ERG2* activity leads to fecosterol accumulation and reduced ergosterol in membranes, thereby decreasing AMB susceptibility [1]. Furthermore, *ERG2* mutations in *N. glabratus* further highlight ergosterol pathway disruption as a critical and clinically relevant resistance mechanism [47].

Concerning the azole resistance in *Candida* species, particularly in clinical settings where FLZ is a first-line treatment, this study observed a high prevalence of resistance, with 91% of *C. albicans* (20/22) and *C. tropicalis* (10/11) isolates exhibiting MICs ranging from 8 to >64 μg/mL. *N. glabratus* isolates showed a 46.2% resistance rate (6/13), with MICs between 64 and >64 μg/mL. Our findings suggest a possible emergence of higher resistance rates, which may represent higher proportions for these species in this region. Globally, resistance rates vary among *Candida* species. In *C. albicans*, resistance rates range from 6% to over 30%, depending on geographical location and clinical setting [48,49,50].

Functionally, resistance often involves *ERG11*, which encodes lanosterol 14-α-demethylase, a key enzyme in ergosterol biosynthesis. We assessed *ERG11* expression in FLZ-resistant isolates, finding an 18-fold increase in *C. tropicalis*, decreased expression in *N. glabratus* (0.167-fold), and no significant change in *C. albicans*. This gene is frequently overexpressed in *C. albicans*, *C. tropicalis*, and *C. parapsilosis*, and when combined with point mutations such as Y132F and G464S, reduces azole binding affinity, thereby promoting FLZ resistance [38,51,52]. Nonetheless, the literature shows variable results, with some studies reporting no significant differences in *ERG11* expression between susceptible and resistant isolates, indicating that additional regulatory mechanisms may be involved [53,54].

In Mexico, earlier surveillance reported full FLZ susceptibility in *C. albicans*, *C. parapsilosis*, and *C. tropicalis*, with elevated MICs observed only in *N. glabratus*. However, more recent reports describe outbreaks of FLZ-resistant *C. parapsilosis* linked to *ERG11* mutations, particularly Y132F, and isolates of *C. albicans* with reduced azole susceptibility in high-risk patients [55]. In *C. tropicalis*, resistance is driven by *ERG11* mutations and overexpression of efflux pumps such as *MDR1* [39].

*MDR1* gene, encoding a major facilitator efflux pump, significantly reduces intracellular drug accumulation. Its upregulation, often observed alongside *ERG11* overexpression, is linked to elevated FLZ MICs and represents a potent, conserved resistance mechanism [39,56]. In *N. glabratus*, the *MDR1* ortholog *FLR1* encodes a major facilitator superfamily (MFS) transporter that mediates azole resistance, particularly to fluconazole, by the same mechanism [57].

In the efflux pump analysis showed moderate *MDR1* overexpression in *C. albicans* (2-fold), reduced expression in *C. tropicalis* (0.23-fold), and stable *FLR1* expression in *N. glabratus* (1.16-fold). Its expression increases under antifungal stress, supporting adaptive and multidrug-resistant phenotypes [58]. Additionally, *PDR1* mutations, which regulate efflux pump genes, further enhance resistance, explaining the species intrinsic low susceptibility and limited treatment options [50,59,60].

Finally, all tested strains were susceptible to 5-FC (MIC 0.25–4 µg/mL), in contrast to higher resistance rates reported elsewhere. Similarly, Tosrisawatkasem et al. (2025) found all *Candida* isolates from oral candidiasis to be sensitive to 5-FC [61]. Resistance, observed in up to 50% of *C. tropicalis* cases, is often due to nonsense or frameshift mutations in *FCY2*. In *C. albicans*, although resistance is uncommon (<1%), it can reach 10%, typically associated with *FCY2* or clade-specific *FUR1* mutations. Furthermore, elevated MICs in *C. tropicalis* have also been linked to *URA3* alterations [12,62,63].

This study provides a comprehensive analysis of epidemiology, antifungal susceptibility, and gene expression of yeast isolates in a real-world hospital setting, employing complementary methods such as chromogenic medium, MALDI-TOF MS and RT-qPCR. It encompasses multiple species, including common and rare isolates, thereby broadening the understanding of fungal diversity and antifungal resistance in nosocomial infections. The correlation between phenotypic profiles and molecular data reinforces the clinical relevance of the findings. However, the cross-sectional design limits the ability to establish causal relationships between clinical factors and antifungal resistance. The relatively small sample size, particularly for less common species, restricts the generalizability of the results. Additionally, the absence of functional analyses and clinical follow-up prevents confirmation of the direct impact of the observed gene expression on resistance mechanisms and patient outcomes. Future multicenter studies with larger isolated collections and longitudinal follow-up are needed to validate and expand these findings.

## 5. Conclusions

This study provides comprehensive evidence on the clinical, taxonomic, and molecular diversity of *Candida* species in hospitalized patients, highlighting the predominance of *Candida albicans* and the increasing relevance of non-albicans species such as *C. parapsilosis*, *C. tropicalis*, and *Nakaseomyces glabratus*. Antifungal susceptibility profiles revealed significant interspecies variability, with high resistance rates to amphotericin B and fluconazole in specific isolates. Differential regulation of resistance-associated genes (*ERG2*, *ERG11* and *MDR1*) underscores that molecular mechanisms of resistance are species-specific and dependent on the antifungal agent used. These findings emphasize the importance of precise species-level identification and molecular analysis as essential tools to guide targeted antifungal therapy and improve clinical outcomes in *Candida* infections.

## Figures and Tables

**Table 1:** Sociodemographic and clinical characteristics of hospitalized patients with infection by *Candida* spp.

	n = 55
Male gender, n (%)	34 (61.8)
Age (years), mean ± *SD*	59 ± 14
BMI (kg/m^2^), mean ± *SD*	27.6 ± 5.0
Comorbidities	
T2DM, n (%)	22 (40.0)
CKD, n (%)	14 (25.5)
CKD + T2DM, n (%)	8 (14.5)
Negative, n (%)	11 (20.0)
Hospital service requesting culture	
Internal Medicine, n (%)	25 (45.5)
Nephrology, n (%)	17 (30.9)
General Surgery, n (%)	9 (16.4)
Gynecology, n (%)	4 (7.3)
Sample source	
Secretions ^1^, n (%)	27 (49.1)
Fluids ^2^, n (%)	19 (34.5)
Blood culture, n (%)	7 (12.7)
Catheter tips, n (%)	2 (3.6)
Specific diagnosis	
CKD, n (%)	18 (32.7)
Soft tissue infection, n (%)	10 (18.2)
Sepsis, n (%)	10 (18.2)
Genital abscess, n (%)	8 (14.5)
T2DM, n (%)	5 (9.1)
Meningitis, n (%)	4 (7.3)

Qualitative variables are presented as frequencies and percentages; quantitative variables as mean ± standard deviation (*SD*) BMI: Body Mass Index; T2DM: Type 2 Diabetes Mellitus; CKD: Chronic Kidney Disease. ^1^ Abscesses, ulcers, and surgical wounds ^2^ Cerebrospinal, peritoneal dialysis, and ascitic fluids.

**Table 2:** Frequency in the interpretation of antifungal susceptibility among *Candida* species to amphotericin B, fluconazole and 5-fluorocytosine.

Antifungal Agent	Range (Min–Max)	GM MIC (µg/mL)	MIC_50_	MIC_90_	Resistant Isolates (%)			
					S	SSD	I	R
*Candida albicans*, (n = 22)								
Amphotericin B (AMB)	0.5–16.0	1.3	2.0	16.0	17 (77.3)	-	-	5 (22.7)
Fluconazole (FLZ)	2.0–64.0	33.0	64.0	64.0	1 (4.5)	1 (4.5)	-	20 (90.9)
5-Flucytosine (5-FC)	0.125–4.0	0.20	0.25	0.5	22 (100.0)	-	-	0 (0)
*Candida tropicalis*, (n = 11)								
Amphotericin B (AMB)	0.5–16.0	3.8	2.0	16.0	4 (36.4)	-	-	7 (63.9)
Fluconazole (FLZ)	0.5–64.0	26.5	64.0	64.0	1 (9.1)	0 (0)	-	10 (90.9)
5-Flucytosine (5-FC)	0.125–2.0	0.3	0.25	0.5	11 (100.0)	-	-	0 (0)
*Candida parapsilosis*, (n = 11)								
Amphotericin B (AMB)	1.0–4.0	1.7	2.0	2.0	4 (36.4)	-	-	7 (63.9)
Fluconazole (FLZ)	1.0–4.0	2.3	2.0	4.0	8 (72.7)	3 (27.3)	-	0 (0)
5-Flucytosine (5-FC)	0.125–0.5	0.2	0.25	0.5	11 (100.0)	-	-	0 (0)
*Nakaseomyces glabratus*, (n = 13)								
Amphotericin B (AMB)	1.0–8.0	1.8	1.0	8.0	7 (53.8)	-	-	6 (46.2)
Fluconazole (FLZ)	8.0–64.0	30.3	32.0	64.0	0 (0)	7 (53.8)	-	6 (46.2)
5-Flucytosine (5-FC)	0.125–0.5	0.1	0.125	0.125	13 (100.0)	-	-	0 (0)

Categorical variables are expressed as frequencies and percentages. Abbreviations: S, Susceptible; I, Intermediate; R, Resistant; SDD, Susceptible Dose-Dependent; GM, Geometric Mean. Breakpoints were established in accordance with CLSI guidelines. MIC_50_ is the minimum inhibitory concentration that inhibits 50% of tested organisms, and MIC_90_ is the concentration that inhibits 90% of them. Antifungal susceptibility was interpreted according to CLSI M27S, 3rd Edition (2022). Amphotericin B (≤1 µg/mL) and 5-flucytosine (≤25 µg/mL) were assessed using widely accepted thresholds; and fluconazole as susceptible ≤ 8 µg/mL, susceptible dose-dependent 16–32 µg/mL, and resistant ≥ 64 µg/mL.

## Data Availability

The data presented in this study are available on request from the corresponding author due to the privacy policies of the involved healthcare institution, which restrict public access to patient-related information to ensure confidentiality and compliance with ethical and legal regulations.

## Supplementary Materials

The following supporting information can be downloaded at: https://www.mdpi.com/article/10.3390/jof11120895/s1, Table S1: Oligonucleotide primers employed for the detection of antifungal resistance-associated genes *ERG2*, *FKS1*, *ERG11*, and *MDR1*.

## Abbreviations

The following abbreviations are used in this manuscript:

5-FC	5-fluorocytosine
ACT1	β-actin
AMB	Amphotericin B
BMI	Body Mass Index
cDNA	Complementary DNA
CKD	Chronic Kidney Disease
CLSI	Clinical and Laboratory Standards Institute
DM	Diabetes mellitus
DMSO	Dimethyl sulfoxide
DNA	Deoxyribonucleic Acid
ERG	Ergosterol
FCY1	Cytosine deaminase
FCY2	Cytosine permease
FKS	Fungal Kinetics of Synthesis
FLZ	Fluconazole
FUR	Phosphoribosyltransferase
GM	Geometric Mean
h	Hour
HCCA	α-cyano-4-hydroxycinnamic acid
HIV	Human Immunodeficiency Virus
I	Intermediate
ICU	Intensive Care Unit
IMSS	Instituto Mexicano del Seguro Social
MALDI-TOF MS	Matrix-Assisted Laser Desorption/Ionization Time-Of-Flight Mass Spectrometry
MFS	Major Facilitator Superfamily
max	maximum
MDR	Multidrug resistance
MIC	Minimum Inhibitory Concentration
min	minimum
min.	Minutes
MOPS	3-(N-morpholino)propanesulfonic acid
PCR	Polymerase Chain Reaction
R	Resistant
RNA	Ribonucleic Acid
rpm	revolutions per minute
RPMI	Roswell Park Memorial Institute
S	Susceptible
SDB	Sabouraud Dextrose Broth
SDD	Susceptible dose-dependent
T2DM	Type 2 Diabetes Mellitus
URA	Uracil biosynthesis gene
WHO	World Health Organization
