# Supplementary material for: Antifungal Susceptibility and Resistance-Associated Gene Expression in Nosocomial Candida Isolates
Source: J Fungi (Basel). 2025 Dec 18;11(12):895. doi: 10.3390/jof11120895 (PMC12734134; doi:10.3390/jof11120895)
Supplement: Supplementary file 1 [file jof-11-00895-s001.zip › jof-3950408-supplementary.pdf]

Supplementary Table S1. Oligonucleotide primers employed for the detection of antifungal resistance-associated genes *ERG2*, *FKS1*, *ERG11*, and *MDR1*.

**Genes/ *Candida* specie**	**Primers Sequence (5' – 3')**	**PCR products length (pb)**	**Reference**
ERG2 *C. albicans*	F: CAGCAATTGGGACTGAAGGTCATAC<br>R: CGGGAATCAATGCACCAGGATAAG	105	(Yan et al., 2008)
ERG2 *C. tropicalis*	F: TACTGCTCTTGGTACCGAAG<br>R: GGCTTGTAAATTTCCGGCTG	120	Desing
ERG11 *C. albicans*	F: ATTGTTGAAACTGTCATTG<br>R: CCCCTAATAATATACTGATCTG	79	(Lohberger et al., 2014)
ERG11 *N. glabratus*	F: GACGTGAGAAGAACGATATCCA<br>R: ATCAAGACACCAATCAATAGGTT	124	(Khan et al., 2014)
ERG11 *C. tropicalis*	F: TTGCCATTCGGTGGTGGTAG<br>R: ACATCTGGAACCTTATCACCGTT	128	(Paul et al., 2022)
MDR1 *C. albicans*	F: TTACCTGAAACTTTTGGCAAAACA<br>R: ACTTGTGATTCTGTCGTTACCG	84	(Rojas et al., 2020)
FRL1 *N. glabratus*	F: TCTTATTCACGATGCTACAAATTGG<br>R: GAATCACAAGGCCAGCAAAGTT	63	(Pais et al., 2016)
MDR1 *C. tropicalis*	F: GCAGTTACCTCATCTGGAGCA<br>R: GCACCAAACAATGGGAACACA	149	(Paul et al., 2022)
ACT1 *C. albicans*	F: GCATCACACTTTTTACAAT<br>R: AACATAATTTGAGTCATCTTT	114	(Lohberger et al., 2014)
ACT1 *N. glabratus*	F: TTGCCACACGCTATTTTGAG<br>R: ACCATCTGGCAATTCGTAGG	225	(Bhattacharya et al., 2018)
ACT1 *C. tropicalis*	F: GTGTTACCCACGTTGTCCCA<br>R: GCGGTGGTGGAGAAAGTGTA	139	(Rojas et al., 2023)
